# Stafne Bone Cavity: A systematic review

**DOI:** 10.4317/jced.62755

**Published:** 2025-06-01

**Authors:** Paolo Boffano, Muhammad Ruslin

**Affiliations:** 1 University of Eastern Piedmont, Novara, Italy; 2 Department of Oral and Maxillofacial Surgery, Faculty of Dentistry, Hasanuddin University, Makassar, Indonesia

## Abstract

**Background:**

The typical imaging aspect of Stafne Bone Cavities is that of a radiolucent ovoid or round shape image in the posterior mandible below the nerve canal. Anyway, bilobate and bilateral lesions have been reported too, as well as lesions above the inferior alveolar canal. The etiology and pathogenesis are still unknown. The aim of the present article is to review the literature about the current knowledge of this peculiar anatomical condition.

**Material and Methods:**

The current study was a comprehensive systematic review that was conducted by using databases on the following online sites: PubMed, Embase, SCOPUS, Wiley Online Library, and Ovid MEDLINE.

**Results:**

Before applying the filters, 238 publications were identified at first in the considered databases. After the application of the filters, the removal of the duplicates, and the screening process, we ended up with 39 articles that were used in our review. The prevalence of Stafne Bone Cavities oscillates between 0,03% and 3,55%. Mean age ranges between 45,4 and 60,8 years. Males outnumber females, with male:female ratios ranging between 9:4 and 11:0. The most common sites of SBC are observed in the posterior mandible, with body and/or angle regions being the most frequent localization in all studies.

**Discussion:**

A wait-and-see approach in terms of a periodic radiograph is recommended in view of the features of this entity, as in exceptional cases tumors seem to have developed in the invaginated salivary gland tissue.

** Key words:**Stafne Bone Cavities, Stafne bone cyst, diagnosis; anatomy, epidemiology.

## Introduction

The Stafne bone cavity (SBC) is named after Edward C Stafne who first described this anatomical variation in 1942. Several terms have been used to describe such asymptomatic radiolucencies of the mandible: Stafne bone cyst, Stafne bone cavity, lingual mandibular salivary gland depression, latent bone cyst, mandibular embryonic defect, aberrant salivary gland defect, static bone cavity, developmental bone defect of the mandible, lingual cortical mandibular bone defect, submandibular salivary gland inclusion, and combinations of the above (1-43).

SBC has no epithelial lining, so it is defined a pseudocyst.

As SBCs are often asymptomatic, in most cases they are incidentally diagnosed on panoramic views during routine performance. The typical imaging aspect of SBC is that of a radiolucent ovoid or round shape image in the posterior mandible below the nerve canal. Anyway, bilobate and bilateral lesions have been reported too, as well as lesions above the inferior alveolar canal.

The etiology and pathogenesis are still unknown, (1-10,23-32).

The differential diagnosis of SBC includes benign lesions, such as traumatic bone cyst, simple bone cyst, dentigerous cyst, odontogenic keratocyst, nonossifying fibroma, or fibrous dysplasia, but also more insidious lesions, such as ameloblastomas, or even malignant lesions, such as metastases (16-28,35-43).

The aim of the present article is to review the literature about the current knowledge of this peculiar anatomical condition.

## Material and Methods

The current study was a comprehensive systematic review that was conducted by using databases on the following online sites: PubMed, Embase, SCOPUS, Wiley Online Library, and Ovid MEDLINE.

The databases were searched for articles published in the English language using the keywords “Stafne bone cyst”, “Stafne bone cavity”, “lingual mandibular salivary gland depression”, “latent bone cyst”, “mandibular embryonic defect”, “aberrant salivary gland defect”, “static bone cavity”, “developmental bone defect of the mandible”, “lingual cortical mandibular bone defect”, “submandibular salivary gland inclusion”, using Boolean operator “OR.”

Duplicate studies were removed. Reference lists from the selected articles were also checked to increase the validity of the search.

As for eligibility criteria, all articles about SBC epidemiology and all articles about SBC clinical and/or radiological characteristics were included. The exclusion criteria included non English language studies, case reports, case series, letters to the editor, biomechanical studies, animal studies, and expert opinions. Research matching the keywords was revised. We used a Microsoft Excel sheet (Microsoft Corporation, Redmond, WA) to complete the selected data.

## Results

Before applying the filters, 238 publications were identified at first in the considered databases.

Before screening, 69 duplicate records were removed.

**Figure 1:**
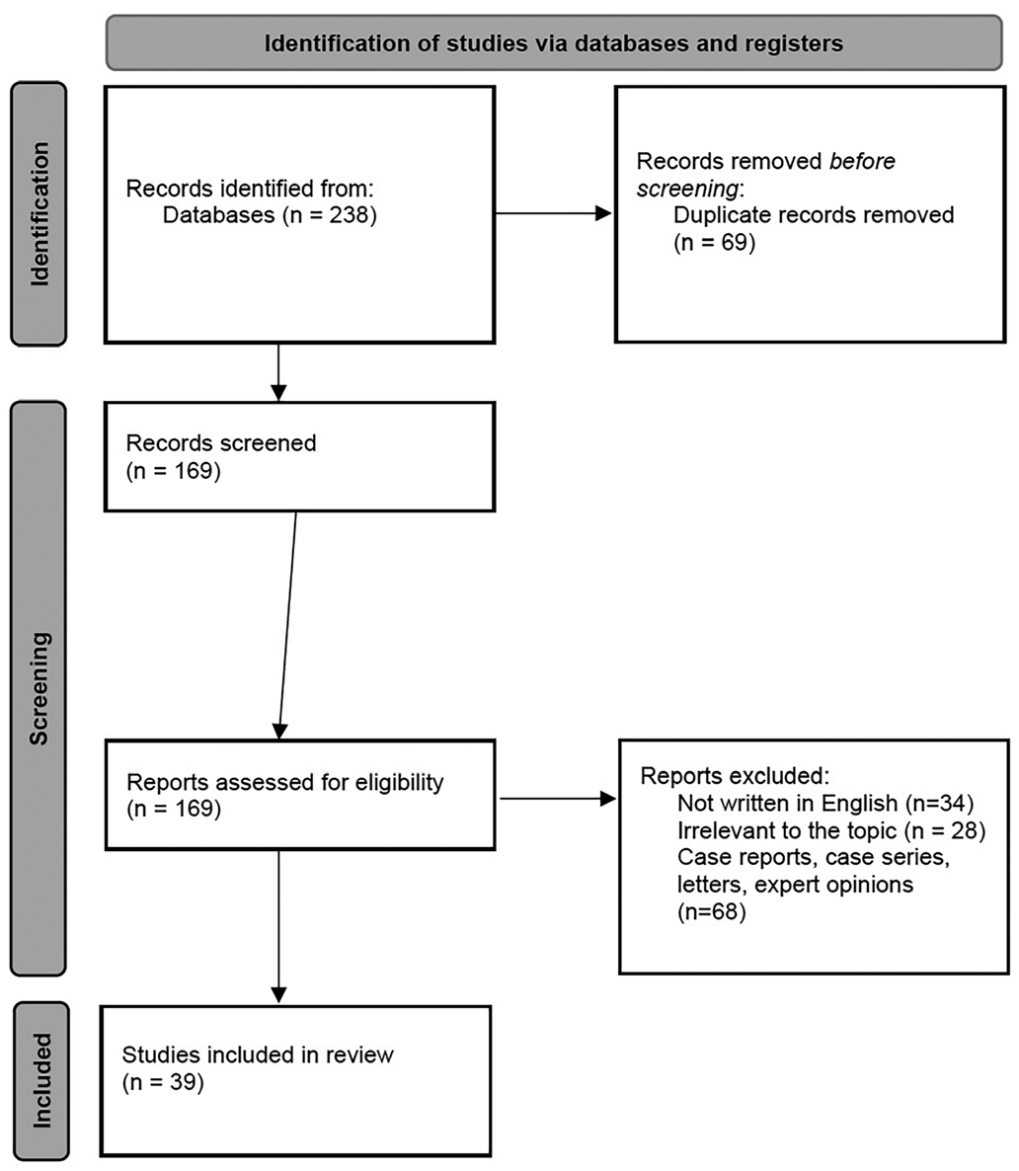
Search flow diagram.

Therefore, 169 publications were screened and assessed for eligibility. We excluded 34 articles that were not written in English. We further excluded 28 articles irrelevant to the topic, and 68 letters to the editor, case reports, biomechanical studies, animal studies, and expert opinions. After the application of the filters, the removal of the duplicates, and the screening process, we ended up with 39 articles that were used in our review (1-39). The screening process is explained in the following search flow diagram depicted in Figure 1.

The included articles about SBC epidemiology are listed in  Table 1.

The included articles about SBC clinical and/or radiological characteristics are listed in  Table 2.

## Discussion

The initial term of SBCs was “Stafne cyst” that reflected the initial description on conventional X-ray studies, although they are not truly cysts. In fact, fluid contents have never been reported in the literature. A more accurate term is Stafne bone cavity (SBC) (1-43).

The diagnosis of SBCs is established when a corticate radiolucency is observed in the characteristic site. Some diagnostic criteria have been identified to establish a correct diagnosis of SBCs: as for the shape, SBCs are usually well-defined round or ovoid radiolucencies with a diameter of 1-3 cm; axially, an opening in the lingual margin of the mandible is usually observed; coronally, an opening in the lingual cortex of the mandible caudal to the mandibular canal is a usual finding; finally, sagittally, SBCs are often encountered in the angle of the mandible in the region of the antegonial notch and submandibular gland fossa (1-32).

Anyway, as aforementioned, variations in the presentation of SBCs have been reported, such as multiple lesions, lesions above the mandibular canal, or bilobated/trilobated lesions.

The etiology and pathogenesis are still unclear and controversial. Different theories have been proposed, including an incomplete calcification of the Meckel cartilage during ossification, the trapping of the submandibular gland during ossification or trauma, the compression by the submandibular gland, the compression from a vascular injury or a vascular-hypertensive aneurysm, and a congenital or developmental cause (1-41).

The hypotheses of the trapping of the submandibular gland during ossification or trauma and the compression by the submandibular gland have been supported by the usual content of SBCs as salivary gland tissues in most studies. According to this theory, a bony resorption would be determined with a consequent invagination of salivary gland tissue into the cavity. Nevertheless, in addition to salivary gland tissue, other tissues, such as muscle, fat, connective tissue, lymphatic, and blood vessels have been encountered within the SBCs, as well as the absence of any tissue. Therefore, such findings represent arguments against both the salivary gland trapping/compression hypothesis and the vascular compression theory, so that the eventual involved tissue would only passively fill up the available space to its anatomical boundaries with-out being responsible for their formation in a first instance. Finally, the congenital/developmental hypothesis seems to be inadequate too, as it is not supported by the high mean age at diagnosis (1-24,32-36).

As for the epidemiology of SBCs, the present review highlights that the prevalence oscillates between 0,03% and 3,55%. The difference of methods used by the single authors (panoramic radiograph, CT, MR) to analyze the presence of SBCs does not seem to be effective to obtain a clear variation in the prevalence of this anatomical condition (1-39).

**Figure 2:**
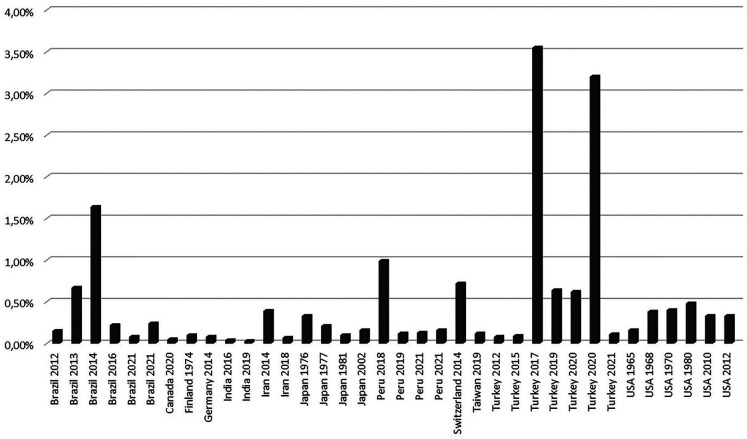
Incidence of Stafne bone cavity diagnosis in the literature.

Figure 2 allows to clearly identify that SBCs have been more frequently diagnosed in Turkey, Brazil, and Peru study populations.

The present review allows to identify that most SBCs are diagnosed in men between 40 and 65 years old. In fact, mean age ranges between 45,4 and 60,8 years. In the included study, males outnumbered females, with male:female rations ranging between 9:4 and 11:0. The present review confirms that the most common sites of SBC are observed in the posterior mandible, with body and/or angle regions being the most frequent localization in all studies. The most frequently encountered shape of SBCs is the oval shape.

Of course, the increasing use of three-dimensional imaging techniques may probably raise the radiological prevalence, thus allowing to discover any lingual impression or indentation. Following a suspect at panoramic radiograph view, preference should be given to cone-beam CT, due to the lower radiation dose. In the past decades, sialography has also been proposed as a diagnostic method, in spite of its technical difficulty, the exposure to radiation, and its stressful feature for the patient. Likewise, open surgical exploration has been proposed too. Nowadays, both sialography and open surgical exploration can be avoided by a thorough radiological diagnosis by panoramic radiograph, Cone Beam CT, CT, and/or MR. Compared to CT, MRI involves no radiation exposure and it presents a higher histological resolution, thus allowing to identify the tissue filling the cavity in the lingual side of the mandible. Therefore, in cases of uncertainty, combining CT and MRI improves the reliability in diagnosing a Stafne bone defect (1-39).

In particular, the differential diagnosis of SBCs should include traumatic bone cyst, simple bone cyst, dentigerous cyst, nonossifying fibroma, odontogenic keratocyst, basal cell nevus syndrome, fibrous dysplasia, ameloblastoma, vascular malformation, giant cell tumor, metastasis, and the brown tumor of hyperparathyroidism (1-42).

## Conclusions

A wait-and-see approach in terms of a periodic radiograph is recommended in view of the features of this entity, as in exceptional cases tumors seem to have developed in the invaginated salivary gland tissue. Anyway, SBCs do not need an aggressive treatment in the absence of functional disorders or subjective symptoms.

## Figures and Tables

**Table 1:** Review of the literature regarding the prevalence of SBCs.

Year	Authors	Country	Number of analyzed cases	Number of SBCs	Prevalence (%)
1965	Lilly et al. (1)	USA	1283	2	0,16%
1968	Karmiol and Walsh (2)	USA	4693	18	0,38%
1970	Johnson (3)	USA	2486	10	0,40%
1974	Oikarinen and Julku (4)	Finland	10000	10	0,10%
1976	Uemura et al. (5)	Japan	3000	10	0,33%
1977	Ehara et al. (6)	Japan	10000	21	0,21%
1980	Correll et al. (7)	USA	2693	13	0,48%
1981	Chen and Ohba (8)	Japan	23000	24	0,10%
2002	Phillipsen et al. (9)	Japan	42600	69	0,16%
2010	Thaw (10)	USA	300	1	0,33%
2012	Sisman et al. (11)	Turkey	34221	29	0,08%
2012	Price et al. (12)	USA	300	1	0,33%
2012	Leonardo et al. (13)	Brazil	667	1	0,15%
2013	Mourao et al. (14)	Brazil	3000	20	0,67%
2014	Schneider et al. (15)	Switzerland	2928	21	0,72%
2014	Assaf et al. (16)	Germany	14005	11	0,08%
2014	De Andrade Salgado et al. (17)	Brazil	1344	22	1,64%
2014	Khojastepour et al. (18)	Iran	773	3	0,39%
2015	Avsever et al. (19)	Turkey	14058	13	0,09%
2016	Goyal et al. (20)	India	6780	3	0,04%
2016	Araujo et al. (21)	Brazil	450	1	0,22%
2017	Demiralp et al. (22)	Turkey	169	6	3,55%
2018	Vaezi et al. (23)	Iran	30000	30	0,08%
2018	Cancho (24)	Peru	1308	13	0,99%
2019	Chen et al. (25)	Taiwan	4000	5	0,12%
2019	Arya et al. (26)	India	18040	6	0,03%
2019	Kurbanova et al. (27)	Turkey	3141	20	0,64%
2019	Chauca (28)	Peru	800	1	0,12
2020	Koc et al. (29)	Turkey	2401	15	0,62%
2020	Sahin and Ozdede (30)	Turkey	189	6	3,2%
2020	MacDonald and Yu (31)	Canada	6252	3	0,05%
2021	Cavalcante et al. (32)	Brazil	17180	15	0,08%
2021	Evirgen et al. (33)	Turkey	33708	39	0,11%
2021	Rodrigues et al. (34)	Brazil	840	2	0,24%
2021	Estrella and Romero (35)	Peru	17875	24	0,13%
2021	Picho (36)	Peru	2521	4	0,16%

**Table 2:** Review of the literature of clinical studies regarding SBCs since 2010.

Year	Authors	Number of SBDs	M, F	Mean age (years)	Locations	Form
2012	Sisman et al. (11)	29	25 M 4 F	49,6	28 body 1 symphyseal region	
2013	Mourao et al. (14)	20	14 M 6 F	51,5	3 angle 15 body 2 symphyseal	19 oval 1 round
2014	Schneider et al. (15)	21	14 M 7 F	53	21 body	
2014	Assaf et al. (16)	11	11 M	58,1	3 angle 8 body	7 oval 4 round
2014	De Andrade Salgado et al. (17)	22	19 M 3 F	-	-	-
2015	Avsever et al. (19)	13	9 M 4 F	49,2	13 posterior	
2018	Vaezi et al. (23)	30	26 M 4 F	45,4	12 angle 18 body	22 oval 8 round
2019	Hisatomi et al. (37)	91	72 M 19 F	60,8	53 angle 36 body 2 symphyseal region	54 oval 37 round
2020	Koc et al. (29)	15	14 M 1 F	58,1	14 posterior (angle/body) 1 symphyseal	
2020	Friedrich et al. (38)	21	19 M 2 F	54,9	18 angle 1 body 1 ramus 1 symphyseal region	
2021	Cavalcante et al. (32)	15	12 M 3 F	49,2	4 angle 10 body 1 ramus	12 oval 3 round
2021	Morita et al. (39)	40	28 M 12 F	57,3	40 posterior (angle/body)	28 oval 12 round

## Data Availability

Availability of data and materials Not available, unless upon request, for privacy reasons.
